# Asynchronous effects of heat stress on growth rates of massive corals and damselfish in the Red Sea

**DOI:** 10.1371/journal.pone.0316247

**Published:** 2025-01-14

**Authors:** Fiza Zahid, Laura Gajdzik, Keith E. Korsmeyer, Jordyn D. Cotton, Daren J. Coker, Michael L. Berumen, Thomas M. DeCarlo

**Affiliations:** 1 College of Natural and Computational Sciences, Hawai‘i Pacific University, Honolulu, HI, United States of America; 2 Cooperative Institute for Marine and Atmospheric Research, University of Hawai‘i, Honolulu, HI, United States of America; 3 Red Sea Research Center, Division of Biological and Environmental Science and Engineering, King Abdullah University of Science and Technology (KAUST), Thuwal, Saudi Arabia; 4 Department of Earth and Environmental Sciences, Tulane University, New Orleans, LA, United States of America; Living Oceans Foundation, TAIWAN

## Abstract

Climate change is imposing multiple stressors on marine life, leading to a restructuring of ecological communities as species exhibit differential sensitivities to these stressors. With the ocean warming and wind patterns shifting, processes that drive thermal variations in coastal regions, such as marine heatwaves and upwelling events, can change in frequency, timing, duration, and severity. These changes in environmental parameters can physiologically impact organisms residing in these habitats. Here, we investigate the synchrony of coral and reef fish responses to environmental disturbance in the Red Sea, including an unprecedented combination of heat stress and upwelling that led to mass coral bleaching in 2015. We developed cross-dated growth chronologies from otoliths of 156 individuals of two planktivorous damselfish species, *Pomacentrus sulfureus* and *Amblyglyphidodon flavilatus*, and from skeletal cores of 48 *Porites* spp. coral colonies. During and immediately after the 2015 upwelling and bleaching event, damselfishes exhibited a positive growth anomaly but corals displayed reduced growth. Yet, after 2015–2016, these patterns were reversed with damselfishes showing a decline in growth and corals rebounding to pre-disturbance growth rates. Our results reveal an asynchronous response between corals and reef fish, with corals succumbing to the direct effects of heat stress, and then quickly recovering when the heat stress subsided—at least, for those corals that survived the bleaching event. Conversely, damselfish growth temporarily benefited from the events of 2015, potentially due to the increased metabolic demand from increased temperature and increased food supply from the upwelling event, before declining over four years, possibly related to indirect effects associated with habitat degradation following coral mortality. Overall, our study highlights the increasingly complex, often asynchronous, ecological ramifications of climate extremes on the diverse species assemblages of coral reefs.

## Introduction

Anthropogenic climate change is becoming an increasingly important stressor for many ecosystems, including coral reefs [1–3]. The excess anthropogenic carbon in the atmosphere, along with additional greenhouse gases, causes global warming, which in turn, increases the frequency of marine heatwaves, the primary driver of coral “bleaching” events [4, 5]. During bleaching, corals become stressed and expel their pigmented symbiotic algae (Symbiodiniceae), turning white in color [6]. After the stressor subsides, corals that are still alive may recover from bleaching by acquiring new, or re-establishing existing, symbiotic algae populations [7]. However, prolonged bleaching often leads to mass coral mortality, which can negatively impact coral reef structural habitat and diversity [8–10].

Coral bleaching and subsequent mortality, resulting from a lack of symbiotic algae population, impacts the environment not only by causing phase shifts toward algae-dominated habitat and reduced structural complexity [5, 11], but also by changing the composition of assemblages that rely on live coral habitats. The general trend is that densities of live coral-dwelling and coral-feeding fish species, including but not limited to damselfishes and butterflyfishes, tend to decrease after severe coral loss, while other groups (especially herbivores such as parrotfishes and surgeonfishes) tend to respond positively by increasing abundance and biomass [10, 12–14]. For example, densities of adult lemon damselfish (*Pomacentrus moluccensis*) were shown to decline in bleached-coral sites in the southern Great Barrier Reef [15], potentially because of a loss in camouflage in bleached corals and refuge spaces within the colonies that makes *P*. *moluccensis* more vulnerable to predation [16–19]. Additionally, metabolic activity in ectotherms has been shown to increase with warmer temperatures, such as during bleaching events, which might suggest a need for a larger food supply or food of higher quality [20–22]. For example, the cholesterol metabolism and uptake of oxygen in *P*. *moluccensis* are activated during heat stress [23, 24]. Similarly, parrotfishes increase their grazing activity on algae under anomalously high temperatures, in turn boosting their growth rate [12, 25]. Thus, reef fish taxa respond differently to disturbance stemming from variations in their metabolism, habitat requirements and behavior (*e*.*g*., live obligate coral dwellers, territorial), and diet preferences (*e*.*g*., omnivores, corallivores, herbivores) [10, 13].

**Fig 1:**
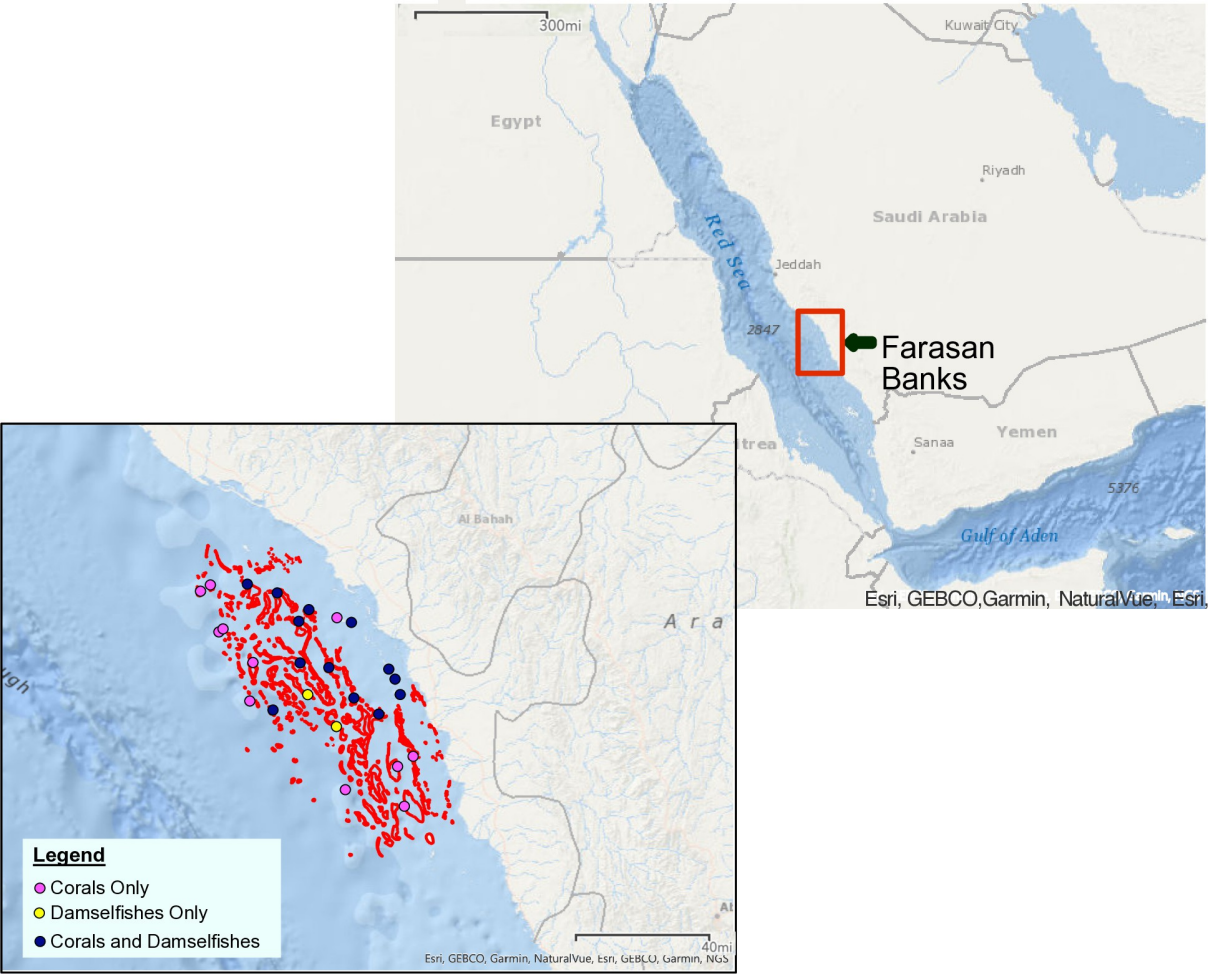
Map of the Farasan Banks located in the central-southern Red Sea. The inset map displays all the sample sites where coral (pink), damselfish (yellow) and both taxa (navy blue) were sampled in 2019. The red polygons behind the sample dots outline coral locations in the region. Bathymetry is represented by the grey numbers in meters.

Our study focuses on the synchrony of growth-related impacts between massive corals and damselfish during and after an upwelling event and a widespread coral bleaching event that occurred within months of each other in the Farasan Banks region of the Red Sea (Fig 1). While damselfish do not often reside on massive *Porites* corals, the inclusion of the widespread *Porites* spp. colonies in this study allows observations of how different taxa in the ecosystem are impacted after environmental disturbances. The first evidence of coral bleaching in the Farasan Banks during 2015 was from a single site in the northern Farasan Banks that showed nearly the entire coral community was bleached during October 2015 [26], and in some areas the bleaching persisted into the beginning of 2016 [27]. Additionally, a broad comparison of benthic surveys conducted before and after 2015, but not during bleaching, revealed that coral cover decreased from 25 ± 17% to 14 ± 12% (mean ± standard deviation) at the Farasan Banks, between the years 2014 and 2019 [28]. Finally, coral skeletal cores collected from long-lived *Porites* spp. colonies in 2019 presented clear evidence of widespread bleaching in the Farasan Banks during 2015, as indicated by anomalous high-density “stress bands” that form during bleaching [29]. These cores also demonstrated that the coral bleaching response was disproportionally high relative to the coral sensitivity to prior heatwaves, likely due to the combined stress in 2015 of high nutrients from upwelling followed by rapid heating [29]. Together, the skeletal-core and benthic-survey data indicate that the 2015 bleaching event in the Farasan Banks (i) was unprecedented in severity over recent decades and (ii) caused widespread mortality of corals. The 2015 bleaching event not only impacted the Farasan Banks, but also other reefs across the tropics. The extremely high temperatures during 2015 to 2016 triggered catastrophic bleaching events around the globe, including the Great Barrier Reef where the reefs underwent a higher bleaching response compared to earlier bleaching events [5]. The coral loss that occurred in the Farasan Banks during 2015 and the 55% increase in algal cover in the following years [28] appears to be of sufficient extent and severity to have potentially impacted reef-associated fish [10].

Despite the documented coral bleaching effects on reef fish at an assemblage level (*e*.*g*., declines in abundance and diversity; [10, 15, 30]), the effects on an individual level—especially related to growth rate—remain largely unexplored. Additionally, while many studies have separately investigated either coral or reef-fish growth responses to disturbance events (*e*.*g*., [12, 31]), no study has addressed the synchrony between the two *in situ*. Here, we examine growth rates recorded in the banding patterns of ear bones, or otoliths, and in coral cores, to measure the effect of the 2015 bleaching event in the Red Sea on massive *Porites* spp. corals and two damselfish species: *Pomacentrus sulfureus* and *Amblyglyphidodon flavilatus* (family Pomacentridae). These two fish species were chosen due to their common presence throughout the Farasan Banks and the representation of two different feeding guilds and habitat specializations that may influence their response to the bleaching and coral mortality. *P*. *sulfureus* is either considered to be a benthic feeder (strictly grazing on algae) or an intermediate feeder (feeding on both animal and algae throughout the bentho-pelagic compartment) [32–34]. Although *P*. *sulfureus* has been observed to occupy large territory on coral rubble at an adult stage [32, 33], this species is also commonly referred to as an obligate live-coral dweller (>80% reliance on corals; [35]), probably because juveniles favor branching coral habitats. In contrast, *A*. *flavilatus* primarily feeds on zooplankton in the water column and has no documented obligate dependence on corals [32, 33, 35]. This characteristic potentially makes the species less likely to be vulnerable to variations in live coral cover and habitat degradation compared to *P*. *sulfureus*. In addition to reef-associated fish species, our study focused on massive *Porites* spp. colonies because they are long-lived and have clear annual density bands, which captures valuable climate information [29], and they record bleaching histories in their skeletons via anomalous annual density bands that are generally representative of coral community-level bleaching responses [36, 37]. We compare growth trends of the *Porites* spp. coral and the two fish species to address the synchrony of responses to—and recovery from—environmental disturbances, such as upwelling, heat stress, and coral mortality, to demonstrate how climate change impacts different aspects of a coral reef ecosystem. While growth rates of the different taxa have been observed in separate studies, they have not been observed simultaneously in one study in which responses to the same environmental drivers can be compared. Fish otoliths and coral cores offer valuable information on biological responses to environmental variability, and our study offers a rare opportunity to quantify interannual, multi-taxa impacts of a marine heatwave in a coral reef ecosystem.

## Methods

### Environmental setting and species sampled

The Red Sea, a semi-enclosed basin, is home to diverse and unique coral reefs that host many endemic species [38, 39]. These reefs exist in a unique range of environmental settings established by the physical processes that occur in the region [38]. Due to the distance from the Gulf of Aden, nutrient concentrations and temperature increase from the northern to southern Red Sea while salinity decreases [40, 41]. There is little terrestrial runoff, so the dominant source of nutrients entering this system comes from the shallow and narrow entrance of the Gulf of Aden [42]. In the summer, due to monsoon wind reversals, a subsurface, high-nutrient water mass called the Gulf of Aden Intermediate Water (GAIW) enters the southern Red Sea [42]. The summer monsoon winds blowing to the southeast also induce upwelling of GAIW along the eastern shelf of the Red Sea due to Ekman transport directed offshore, including the extensive coral reef area of the Farasan Banks, located in the central-southern Red Sea [42]. Maximum temperatures in the Farasan Banks occur after the monsoon winds weaken in late summer and upwelling ceases [43]. As a result, the degree of early-summer upwelling (June-July) occurs independently of the late-summer (September or October) peaks in temperature [29, 43].

The Farasan Banks are composed of hundreds of reefs ranging from inshore to offshore environments, including different reef formations (*e*.*g*., fringing reefs around islands to atolls). Sampling in this study was conducted across the shelf, from nearshore turbid reefs with adjacent mangroves, to midshelf reefs, and shelf-edge atolls. Both damselfish and coral cores were collected at the same sites, whenever possible. However, at some reef sites we did not find one or both species of damselfish, and at some other sites we did not find living *Porites* colonies. In total 2 sites contained only damselfishes, 12 sites contained only corals, and 13 sites contained both taxa. Some of the same reefs sampled in the present study were previously monitored with temperature loggers placed to record upwelling-associated cooling events by [43] and where *Porites* spp. coral cores were taken for stress band analysis in 2019 [29], which included several massive-morphology species (*P*. *lutea*, *P*. *lobata*, and *P*. *solida*). These coral cores from [29] were used for analysis in our study. Approximately 60 cores were taken by [29] using an underwater pneumatic drill with a 5 cm diameter diamond-impregnated bit [29]. We collected 179 *P*. *sulfureus* and 130 *A*. *flavilatus* individuals from between 1 and 10 meters depth at 15 coral reef locations in the Farasan Banks region of the Saudi Arabian Red Sea between April and May of 2019 (Fig 1). Standard lengths of *P*. *sulfureus* ranged from 3.9 to 7.8 cm with an average of 6.1 cm, and for *A*. *flavilatus* they ranged from 5.5 to 7.5 cm with an average of 6.6 cm. The maximum published lengths for *P*. *sulfureus* were 11 cm and 10 cm for *A*. *flavilatus* [44]. Otolith analysis revealed an age range of 4–16 years, with a median age of 9, for *P*. *sulfureus* and a range of 4–10 years, with a median age of 7, for *A*. *flavilatus*. Sampling for the corals and damselfishes was carried out under approved protocols by both King Abdullah University of Science and Technology’s (KAUST) Biosafety and Ethics Committee and IACUC. Fish were rapidly killed by cervical transection using a sharp knife which was inserted caudal to the skull to sever the spinal cord and cervical vertebrae and was followed by pithing to ensure death. This method complies with the American Veterinary Medical Association (AVMA) Guidelines for the Euthanasia of Animals [45] and is considered humane and painless. Research was carried out under the general auspices of KAUST’s arrangements for marine research with the Saudi Arabian Coast Guard and the Ministry of Environment, Water and Agriculture.

### Otolith analysis

**Fig 2:**
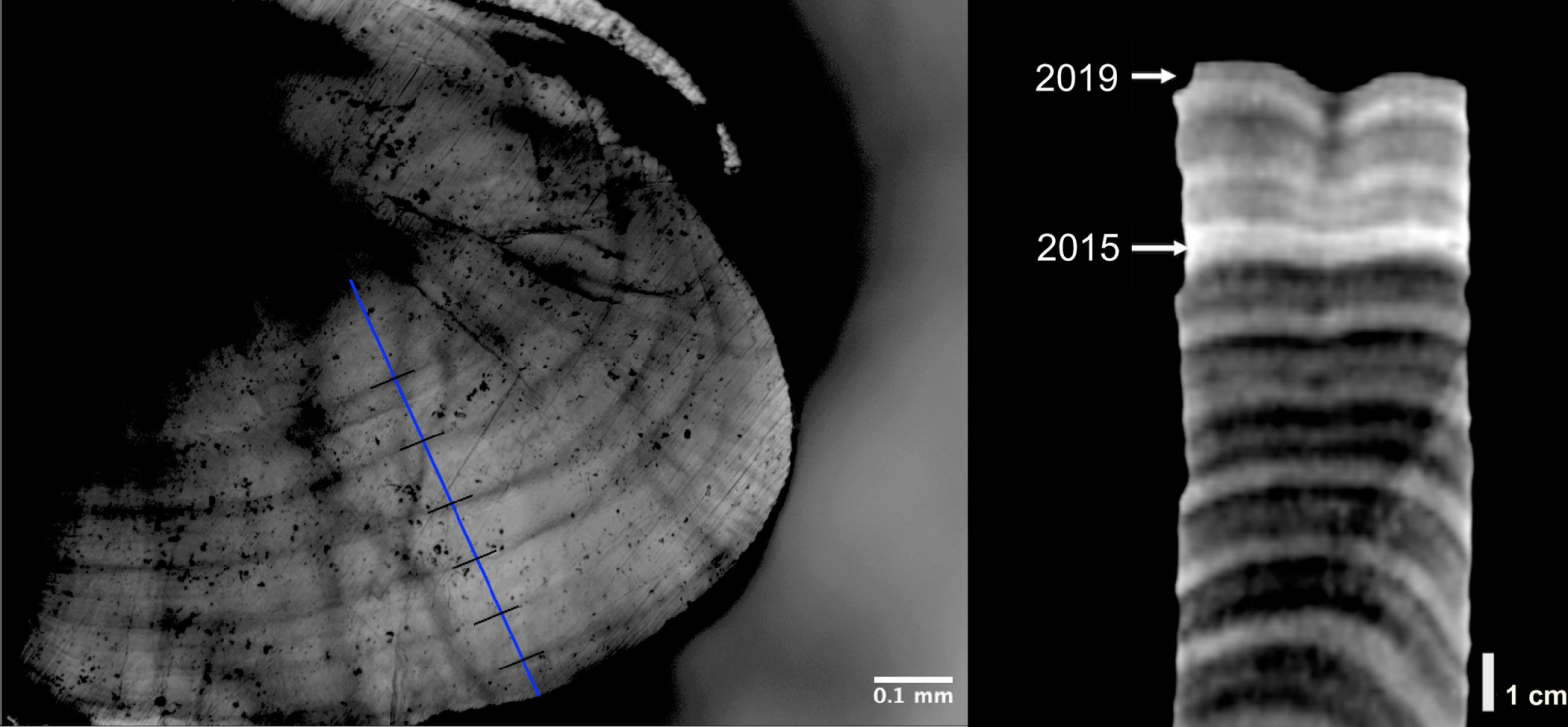
Examples of annual growth increments in an otolith (left) and coral core (right). Left: the otolith is a polished sagittal cross-section from *Pomacentrus sulfureus*. The blue line is drawn perpendicular to the growth lines, and parallel with the axis of growth. The black lines mark the annual increments. Right: a computed tomography (CT) scan of a *Porites* spp. coral from the Farasan Banks with one high density stress band (2015), among the annual density bands ranging from approximately 2009 until date of collection in 2019.

Otoliths (two for most samples) were extracted with a scalpel and stored in absolute ethanol. Prior to processing, each otolith was rinsed for 10 seconds in a 10% bleach solution and then washed with ethanol to remove any residual organic matter. To view the growth increments, we followed procedures from [12], using a standard grinding technique. The otoliths were attached to a glass slide with thermoplastic cement (Crystal Bond). We manually sanded the otolith with 600 to 7000 grit paper with water to produce a transverse thin section of one otolith from each fish. This transverse section enables visualization of an internal surface of the otolith, where the growth increments are often most clearly defined. The first step of the process involved attaching one otolith with the cement to the edge of the slide, such that the transverse section to be visualized was even with the slide edge while half of the otolith extended beyond the slide, and the sulcal ridge was perpendicular to the slide edge, similar to methods by [12]. We then manually ground the half of the otolith extending from the slide edge so that the desired transverse section was exposed. The slide was then reheated to 100°C to melt the thermoplastic cement, and we flipped the otolith over, such that the newly sanded side was flat on the slide. Finally, we manually ground the remaining half of the otolith until it neared the desired transverse section and clear increments were seen under a microscope. The revealed growth increments were visualized under an Olympus CX31 microscope using 10x magnification. It is assumed that otolith growth is correlated with somatic growth [46, 47]. Using a OMAX A3580U microscope digital camera, along with the program *ToupLite*, we collected pictures of the fully polished otoliths. Each fish had one otolith analyzed with at least one set of increment measurements, where the most recent five years or more could be seen (Fig 2). In total, 300 samples were processed, but not all otoliths revealed measurable increments.

The distances between growth increments were measured using *ImageJ* (version 1.53; [48]). These measurements were conducted in a standard way by measuring the distance from the outside edge of one increment to the outside edge of the next perpendicular to the increments. A photo of the scale bar was used to calibrate the distance in *ImageJ* [48]. Afterwards, standard cross-dating statistics were used to compare growth rates among the years using the following R packages: stringi, dplR, graphics and utils [49–52]. The output (1) indicated potential dating errors for re-inspection, (2) provided among-sample correlation statistics, and (3) produced a “master” chronology time series used to track patterns in the growth rates. Along with the master time series, all individual samples went through an autoregressive detrending model with the dplR package to remove the ontogenetic effect of natural otolith width decline with age. If this effect was not removed, the chronology would show the natural decline of growth with age, instead of showing the effect of environmental variation on the damselfish. While different methods have been used for removing ontogenetic growth trends, this step is commonly applied in most otolith studies (*e*.*g*., [12, 53]).

From the 300 samples processed, 217 samples, with visible increments for at least 5 years, proceeded to the cross-dating stage. Some samples were remeasured and some were removed from the dataset if increment counts or measurements were uncertain. From the 217 samples, 57 were removed due to having unclear chronology or poor interseries correlations that hindered confidence in the master time series. Many of the remaining samples were remeasured if there were any doubts in increment identification. For most samples, the start of the chronology was lagged by one year from the collection year—the otoliths were collected in 2019 but otolith increments do not necessarily align with calendar years, which made it difficult to initially determine if the first complete increment represented our nominal year 2019 or 2018. Thus, final decisions for the year assignment of the outer most ring for each otolith were based on a combination of inspection of the proximity of the final increment measurement to the otolith edge, and whether the interseries correlation was higher with, or without, a lag. An improvement suggested a more accurate time series of both the individual and the species. Rather than remeasuring or lagging multiple samples at once, making it harder to determine which changes were beneficial, one to three samples were adjusted at a time. The master time series was updated with these small, controlled changes and the shift in the correlations (both individual interseries and overall correlations) were recorded. At the end, we retained 156 total otoliths (88 from *P*. *sulfureus* and 68 from *A*. *flavilatus*) with confident measurements that were used to produce a master time series for each species. This series was standardized so the expected growth rate was centered at 0, rather than 1 to account for individual variation in growth. Negative values of our standardized growth indicate that growth was anomalously slow during that year, while values above 0 are indicators of higher-than-usual growth. Hereafter, the mention of growth rates refers to this standardization.

### Coral analysis

*Porites* coral cores were previously collected from the Farasan Banks region of the Red Sea and scanned via computed tomography (CT) following the methods outlined in [29]. Here, annual density bands were identified and measured for extension rate using the program coralCT [54]. Briefly, two-dimensional transverse digital slices cut from the full three-dimensional scans were interpreted for alternating high- and low-density bands, a pair of which represents one year of growth (Fig 2). The coralCT program then traces the growth direction in three dimensions through each core while measuring the growth distance between consecutive bands. Coral growth parameters include extension (cm yr^-1^), density (g cm^-3^), and calcification (g cm^-2^ yr^-1^), with calcification being the product of density and extension. We used the extension measurements as these are comparable with otolith increment widths, although calcification data produced similar results (S3 Fig), and the choice does not affect our conclusions. Crossdating for the corals using the measurements from coralCT followed the same methodology as the otolith crossdating in R.

### Statistical analysis

A series of linear marginal models were constructed to test for the effects of various factors on the standardized growth rate of the fish species. A marginal model was used to account for the repeated-measures on the fish samples, but because growth rates were standardized, we examined the average population trend and not individual differences that would require random effects [55]. The factors examined included standard length, upwelling index, mean annual temperature, distance from shore, mean summer temperature, years since the bleaching event of 2015, and degree heating weeks (DHW) to explain the changes in growth rates in damselfishes. Due to missing standard lengths in the dataset, four samples were removed. The years since the bleaching event factor was included to account for longer-term impacts on growth not represented by the environmental factors. Mean annual temperature, along with mean summer temperature and DHW, were calculated with sea surface temperature (SST) data retrieved from the National Oceanic and Atmospheric Administration (NOAA) Optimum Interpolation Sea Surface Temperature product (OI-SSTv2). Upwelling index was defined as the difference between the June maximum and the August minimum SST, based on [29], which provides an indication of the degree of upwelling where higher values relate to stronger upwelling. We calculated the mean summer SST based on July to October because the maximum SST often occurs as late as October in this area [43]. Degree heating weeks were defined as hotspots, where SST was greater than 1°C above the maximum monthly mean, over a defined period of time (12 weeks) [56]. Because the interseries correlation of the combined species data was similar to the correlations of the two individual species (0.544 for *P*. *sulfureus*, 0.573 for *A*. *flavilatus*, and 0.553 for combined), the species were combined for the linear models. We followed a similar approach to the models created by [12] with the exception that species was not added as a random effect due to the standardization of the growth rates, and thus we did not have mixed effects in our model. A step-up approach was used to build the models, starting with simple models using one factor and expanding to more complicated models with combinations of the significant factors. Akaike’s information criterion (AIC) scores using Maximum Likelihood (ML) estimation were compared to identify the best models [55]. Along with these models, Pearson correlations were calculated between all quantitative factors (S1 Table) to determine if any explanatory variables were correlated, which could complicate model interpretations. The marginal models were created using IBM SPSS Statistics (v. 27), with the repeated effect of year with subjects as individual fish samples and first-order autoregressive (AR1) as the covariance structure because it provided the lowest AIC scores. The top two models were rerun with a Restricted Maximum Likelihood (REML) approach to estimate the parameters and significance [55].

The coral chronology was treated similarly to the otolith chronologies. Extension rates were standardized to be centered at zero, and graphed with a 95% confidence interval to enable interpretation of years with growth significantly greater or less than the mean. The coral growth rates did not undergo the autoregressive detrending model, as corals have longer lifespans than the damselfishes and generally do not encounter the natural ontogenetic decline in growth. The coral chronologies mostly extended back several decades, but only the time period overlapping with the otolith chronologies (2010–2018) was examined here. From here on, we use the term “coral growth rates” to refer to these standardized growth rates. Similar to the fish growth rate analyses, marginal models of coral growth rates were examined with fixed effects of mean annual temperature, upwelling index, and DHW. The repeated effect was included with individual coral cores as subjects and an unstructured covariance structure for the residuals.

## Results

### Damselfish growth

**Fig 3:**
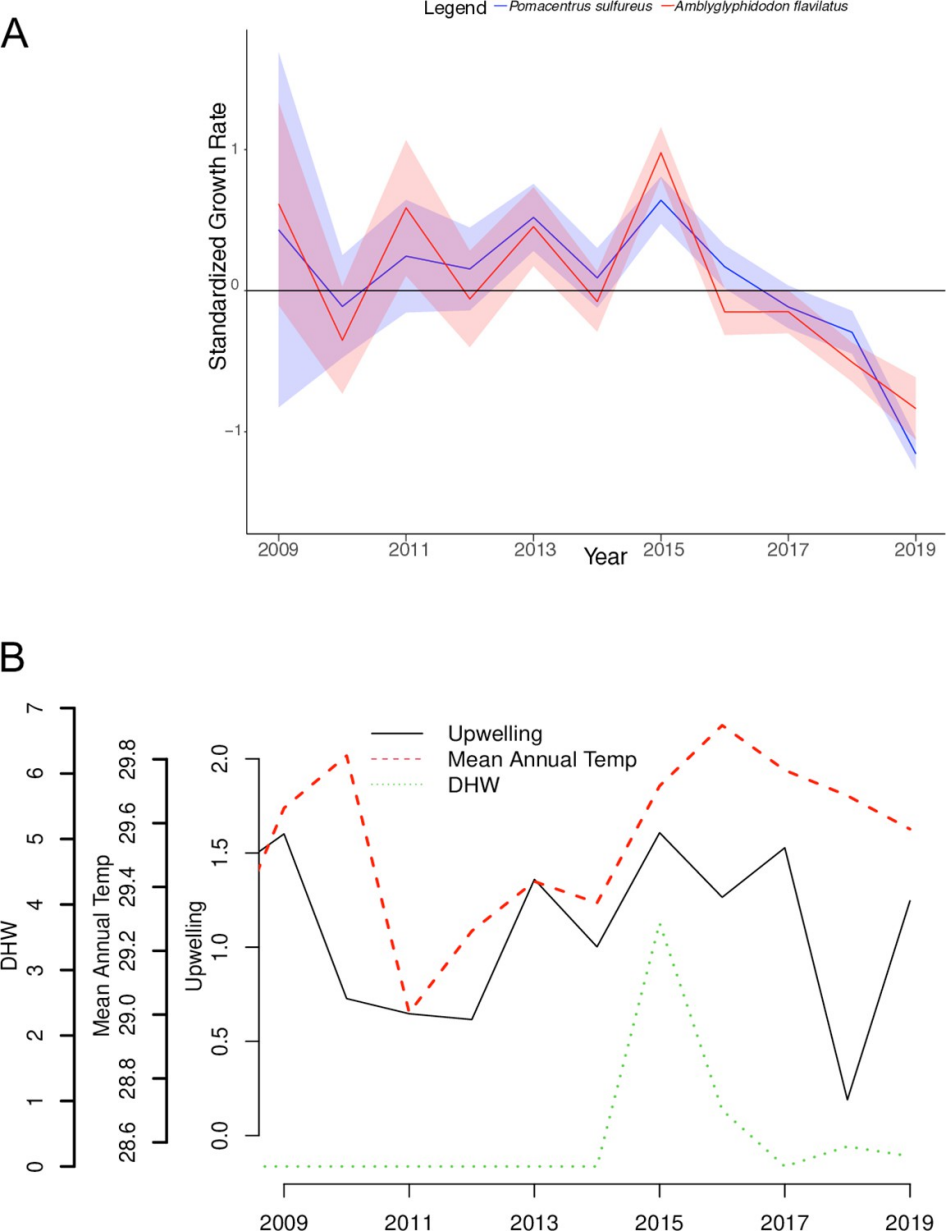
Damselfish growth and environmental variations in the Farasan Banks. (A) Master chronology of growth anomalies for both species, *Pomacentrus sulfureus* (blue) and *Amblyglyphidodon flavilatus* (red), normalized at zero. The growth rates are standardized with units of z-scores. Shaded region represents the 95% confidence interval over the years where multiple fish samples were analyzed. (B) The mean annual temperature (°C), the maximum degree heating weeks (DHW), and the upwelling index for the same time range as the damselfishes at the Farasan Banks.

We started with crossdating for both damselfish species individually. For both *P*. *sulfureus* (n = 88) and *A*. *flavilatus* (n = 68), we found mean interseries correlations of 0.57, which represents the average r value between each individual otolith chronology and the “master” chronology that is effectively the average of all other otoliths. From the cross-dated chronologies, we extracted the time series for each sample that went through the autoregressive detrending model, standardized to center at zero and displayed it graphically with a 95% confidence interval (Fig 3). Both species exhibited a positive growth anomaly during 2015, synchronous with the coral bleaching and upwelling event (Fig 3). In fact, each species’ maximum growth rates occurred in 2015, one of only two years (along with 2013) in which both species had growth rates significantly greater than zero (Fig 3). After 2015, growth rates steadily declined, with the most recent two years (2018 and 2019) being the only years in which both species’ growth rates were significantly less than zero (Fig 3).

Marginal models were created to account for the repeated measures of having multiple growth measurements on each individual otolith (Table 1). We present the two best (lowest AIC, S2 Table) models for comparison, because the ΔAIC between them was very small (1.8). In both models, positive growth was best explained by DHW and years since bleaching. In the second-best model the upwelling index was included as an additional factor. Other factors such as distance from shore and standard length of fish did not improve fit. DHW had a positive impact on growth (Model 1, F_1,1104_ = 46.7, p<0.001), while years since bleaching had a negative effect (Model 1, F_1,647_ = 183.5, p<0.001) (Table 1). The addition of upwelling had a small and not statistically significant effect (Model 2, F_1,783_ = 0.20, p = 0.66) and removing it from the model resulted in only a negligible reduction in AIC, but we present this model in Table 1 for comparison to show the extent of its effect on growth.

**Table 1:** Estimates of fixed effects from the two best marginal models of the damselfishes’ standardized growth rate. The factors include degree heating weeks (DHW), the upwelling index and years since the 2015 bleaching event.

Model	Parameter	Estimate	SE	df	t	p-value
**1**	Intercept	1.08	0.016	666.3	69.4	< 0.001
	DHW	0.058	0.009	1104	6.83	< 0.001
	Years Since Bleaching	-0.099	0.007	647.3	-13.5	< 0.001
**2**	Intercept	1.07	0.031	1048	34.9	<0.001
	DHW	0.057	0.009	1054	6.14	<0.001
	Years Since Bleaching	-0.098	0.007	684.0	-13.2	<0.001
	Upwelling	0.011	0.025	782.6	0.442	0.66

### Coral growth

**Fig 4:**
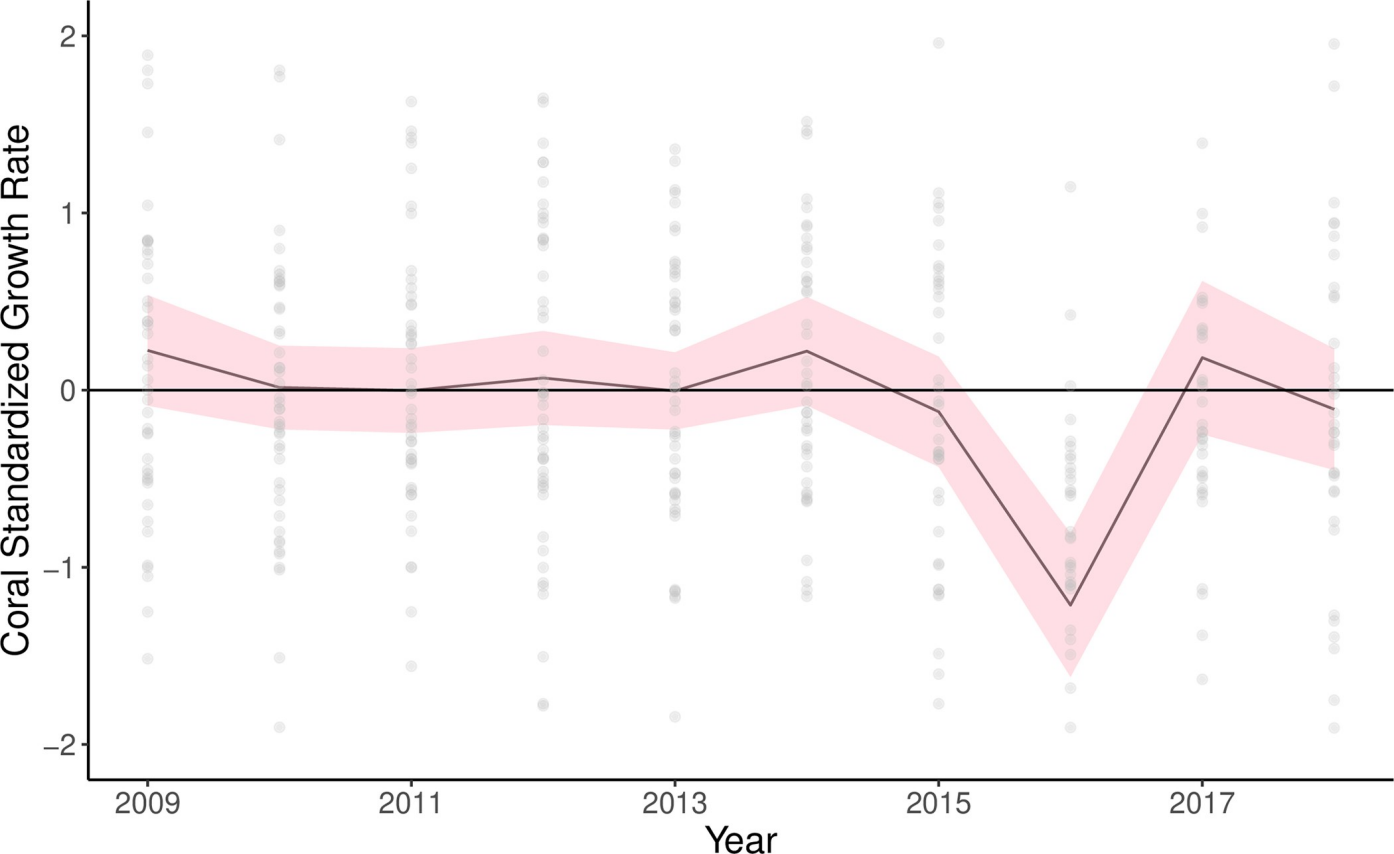
Master chronology of standardized *Porites* coral growth rates from Farasan Banks between 2009 and 2018. The shaded region represents the 95% confidence interval.

The final mean interseries correlation for the *Porites* spp. corals after crossdating was 0.167 (n = 48). The negative growth anomaly occurred in 2016, when growth rate was significantly below zero. The year 2016 was the only year the corals displayed a significant growth anomaly in the entire time series (Fig 4). The best linear marginal model (lowest AIC score) for explaining negative coral growth rates had only DHW as a fixed effect (F_1,39.9_ = 4.13, p = 0.049) (Table 2). Since the only significant change in growth occurred one year after the bleaching event of 2015, the measured growth responses may not have correlated precisely with the environmental drivers that led to the coral bleaching. Indeed, most of the models were comparable in fit with AIC differences of less than 2 (S2 Table). This result means that we could not conclusively distinguish among the models with various environmental factors as explanatory variables. In the model with only DHW as an explanatory variable, the effect of DHW on coral growth was negative but small (-0.021, SE 0.01).

**Table 2:** Estimates of fixed effects from the best marginal model of the corals’ standardized growth rate. The only factor included is degree heating weeks (DHW).

Parameter	Estimate	SE	df	t	p-value
**Intercept**	-0.037	0.043	47.864	-0.861	0.393
**DHW**	-0.021	0.01	39.906	-2.033	0.049

## Discussion

We combined growth-rate time series for long-lived corals (*Porites* spp.) and two reef fish species (*P*. *sulfureus* and *A*. *flavilatus*) to investigate the synchrony in the response to heat stress between these disparate taxa. Both damselfish species displayed positive growth anomalies in the year 2015 during the heat stress and upwelling event, and then growth declined after 2015 (Fig 3). Our results are consistent with [12]’s study whose results revealed that parrotfishes showed a 35% increase in the growth index during the bleaching year [12]. In our data, growth during the bleaching and upwelling year (2015) was also significantly faster than expected, although the damselfish growth rates steadily declined after 2015 to levels significantly lower than expected, in contrast to [12]. The parrotfishes benefited from new growth of algae on the surface of corals that died from bleaching [12, 26], while our damselfishes might have benefited from increased food sources—including both algae cover and plankton availability—resulting from the nutrient-rich waters upwelled during 2015.

Additionally, we tested if environmental variables could explain the patterns observed in the chronologies of damselfishes, which confirmed that DHW had a significant positive impact on growth (Table 1). Additionally, years that followed the 2015 bleaching event also had a significant negative effect, suggesting that a downward trend of growth rate occurred after 2015. The year 2015 was unusual for this region due to the anomalously strong upwelling event in the early summer, followed by the heat stress event in late summer that sparked coral bleaching and eventually caused a reduction of live coral cover by 44% [29, 30]. Several hypotheses could explain why the damselfish responded with the short-term positive growth anomaly in 2015 and then steadily declined in the years afterwards. As ectotherms, the higher temperatures will raise metabolic rates in reef fishes, which will require a larger food intake [21, 22] which can also accelerate growth rates [57, 58]. Although the model did not find that upwelling had a significant overall effect on growth rates, with the upwelling event in early summer, the damselfish may have accessed an increase in available food, and possibly an increase in food quality, at a time when food demand and the capacity for growth was elevated by the heating event. Upwelling brings nutrient-rich water from greater depths up to the surface, along with inorganic nutrients that increase primary productivity [42, 59], and could lead to higher abundances of zooplankton and other damselfish prey [60]. In other regions of the world, upwelling events have been reported to increase planktivorous reef fish abundances [61]. While upwelling may benefit reef fish, the upwelling event at the Farasan Banks in 2015 was implicated in exacerbating the effect of the late-summer heatwaves on the corals leading to mass bleaching and mortality [29]. Therefore, the increase of potential food supply from the upwelling event might have provided enough energy to support higher metabolic demands of damselfishes during the anomalously warm temperatures in the late summer. This change would result in a temporary increase in growth during 2015, as seen in our results (Fig 3).

Similar to the notion that food supply could modulate growth responses, we expected that larger fish, with presumably lower mass-specific metabolic rates, would prove more resilient to disturbance than smaller conspecifics [22]. Although the length of the damselfish did not improve our model’s AIC values, studies have shown that size can impact metabolism, and in return growth rates of coral fish [22]. Larger fish may also be more susceptible to the effects of increased temperatures on metabolic capacity. For example, larger coral trout (*Plectropomus leopardus)* appear to be more limited in increasing maximal aerobic metabolism with increases in temperature, which could lead to reduced activity levels or energy invested in growth [22]. Additionally, increased feeding rates are required to meet basal metabolic demands at warmer temperatures [21]. If the need for greater food intake is not consistently met, this could explain the lack of a size effect in our study. While individual size was not a significant factor in our study, it could impact metabolic rates, and consequentially growth rates, in reef fish communities in general. For instance, a summer with just a high temperature bleaching event but not upwelling might result in limited food, potentially leading to a more pronounced effect of size on the response of the damselfishes to the heat stress. Thus, the potential for different growth responses to marine heatwaves among size classes of reef fish remains an intriguing hypothesis worthy of future study.

Damselfish growth steadily declined after 2015 (Fig 3), despite the initial positive impact of DHW, which may be due to the long-term effects of coral mortality in the region. [28] observed an overall increase in algal cover and a decrease in live coral cover between 2014 and 2019 at the Farasan Banks. As live coral habitat decreased in the region, it is possible that the damselfish encountered higher than normal inter- and intra-specific competition for the remaining habitat, as observed for others species of damselfish [62, 63]. This increase in competition might deplete the damselfish energy stores, leaving less for growth. Predation pressure has also been shown to decrease damselfish abundances after bleaching events due to disruption of chemical cues that signal predator avoidance behavior [16]. Furthermore, as the corals initially turn white from bleaching, damselfish temporarily lose their camouflage advantage [19]. Reef fish with yellow coloring such as *P*. *sulfureus* and *A*. *flavilatus* are able to blend into live coral habitat but stand out against bleached-white and algal-covered coral, increasing stress levels [17, 18]. Branching corals, which is the habitat damselfishes prefer [32–35], exhibited extensive mortality following the 2015 bleaching event [28]. Moreover, with an increasing algae presence in areas of these bleached or dead branching corals, damselfishes have a diminished ability to hide between the branches for shelter [8]. These stressors related to coral loss could account for the decline in damselfish growth after 2015. After the damselfish initially benefited from the higher temperature and upwelling events of 2015, long-term negative effects of their degrading coral habitat could have diverted their energies more towards finding new shelter, avoiding competition, and predators, than feeding, hence decreasing their growth.

In contrast to the positive growth anomaly in damselfishes, we observed a decline in coral growth rates after the height of the bleaching event in 2015, but mainly manifesting during 2016 (Fig 4). Previous investigations of coral growth anomalies following the major 1998 global bleaching event have shown mixed results. In the Caribbean, growth rate of *Orbicella faveolata* (family, Merulinidae) declined dramatically during the 1998 mass bleaching event [31]. Conversely, in Palau, there were no clear changes in *Porites* growth during the 1998 or 2010 bleaching events [36]. Finally, *Porites* growth rates declined during 1998 at some, but not all, sites studied by [31] on the Great Barrier Reef. Our data show a significant negative anomaly in growth only during 2016, the year after bleaching, in large part due to missing bands. For 15% of cores, there were only two complete high-low density band pairs visible between the 2015 stress band and the collection time in early 2019, indicating the absence of an entire annual density band. Since the 2015 stress band was visible, and this likely formed during heat stress that peaked in October 2015, we assumed calcification ceased during and after heat stress, with the growth ceasing mainly during calendar year 2016. Regardless of whether the missing years were assigned to 2015 or 2016, our data clearly indicate a perturbation toward anomalously low growth during and immediately after the bleaching event, followed by a return to typical growth rates after two years (Fig 4). That we observed a clearer growth rate response to bleaching in *Porites* spp. corals compared to previous studies [31, 36] may reflect a more severe bleaching event, consistent with the remarkably prominent stress bands found in the corals that survived this event (Fig 3; [29]).

Our study reveals that corals and reef fishes can be asynchronously impacted by marine heatwaves and other environmental disturbances. In this case, the damselfishes initially benefited from the high temperatures, and possibly concurrent upwelling, that occurred in 2015, at the same time that heat stress caused mass bleaching and mortality of corals [27–29]. The negative effects of the heat stress were recorded in the high-density stress bands of *Porites* corals, and the corals almost immediately encountered lower growth rates but then quickly recovered by 2017. In contrast, damselfish growth steadily declined in the years after coral mortality, suggesting that these fish species were negatively impacted by the long-term effects of habitat degradation, unlike *Porites* corals. With climate change increasing the frequency and severity of bleaching events [4], damselfishes might not be able to acclimatize to environmental disturbance despite temporary boosts in growth. These negative changes might not be immediately visible in the damselfishes as in the bleached corals but our study shows the complexity of different time scales in the response to environmental disturbances within an ecosystem. Climate change will likely have cascading consequences on not just individuals, but entire coral reef ecosystems like the Farasan Banks, where environmental disturbances such as marine heatwaves and upwelling can impact corals and reef fishes asynchronously.

## Supporting information

S1 Fig Plot of *P*. *sulfureus* samples. Year is represented on the horizontal axis and individual fish sample’s growth rate is represented on each vertical axis labeled by PXXX. Peaks in each growth rate represents variations of growth.The master chronology in the paper combines all of these individual growth rates into something more digestible. Only 46 of the samples are labelled due to space limitations on the Fig.
(TIF)

S2 Fig Plot of *A*. *flavilatus* samples. Year is represented on the horizontal axis and individual fish sample’s growth rate is represented on each vertical axis labeled by AXXX. Peaks in each growth rate represents variations of growth. The master chronology in the paper combines all of these individual growth rates into something more digestible. Only 36 samples are labelled due to the space limitations on the Fig.
(TIF)

S3 Fig Coral standardized calcification rates. Master chronology of standardized *Porites* coral calcification rates from Farasan Banks between 2009 and 2018. The shaded region represents the 95% confidence interval. The grey dots represent individual sample calcification values.
(TIF)

S1 Table Pearson correlation matrix of predictors used in the marginal models. (PDF)

S2 Table Damselfish marginal model outputs (SPSS). Akaike’s information criterion (AIC) scores for tested Marginal Models of the damselfishes’ standardized growth rates between 2010 and 2019, listed from best to worst. All models had a repeated effect of ‘year’ with subject as individual fish samples and first-order autoregressive (AR1) as the covariance structure. To allow for comparison of models with different fixed effects, Maximum Likelihood (ML) estimation was used.
(PDF)

S3 Table Coral marginal model outputs (SPSS). Akaike’s information criterion (AIC) scores for tested Marginal Models of the corals’ standardized growth rates between 2010 and 2018, listed from best to worst. All models had a repeated effect of ‘year’ with subject as individual coral cores and an unstructured covariance matrix. To allow for comparison of models with different fixed effects, Maximum Likelihood (ML) estimation was used.
(PDF)
