# Supplementary figures and images for: Asynchronous effects of heat stress on growth rates of massive corals and damselfish in the Red Sea
Source: PLoS One. 2025 Jan 14;20(1):e0316247. doi: 10.1371/journal.pone.0316247 (PMC11731716; doi:10.1371/journal.pone.0316247)

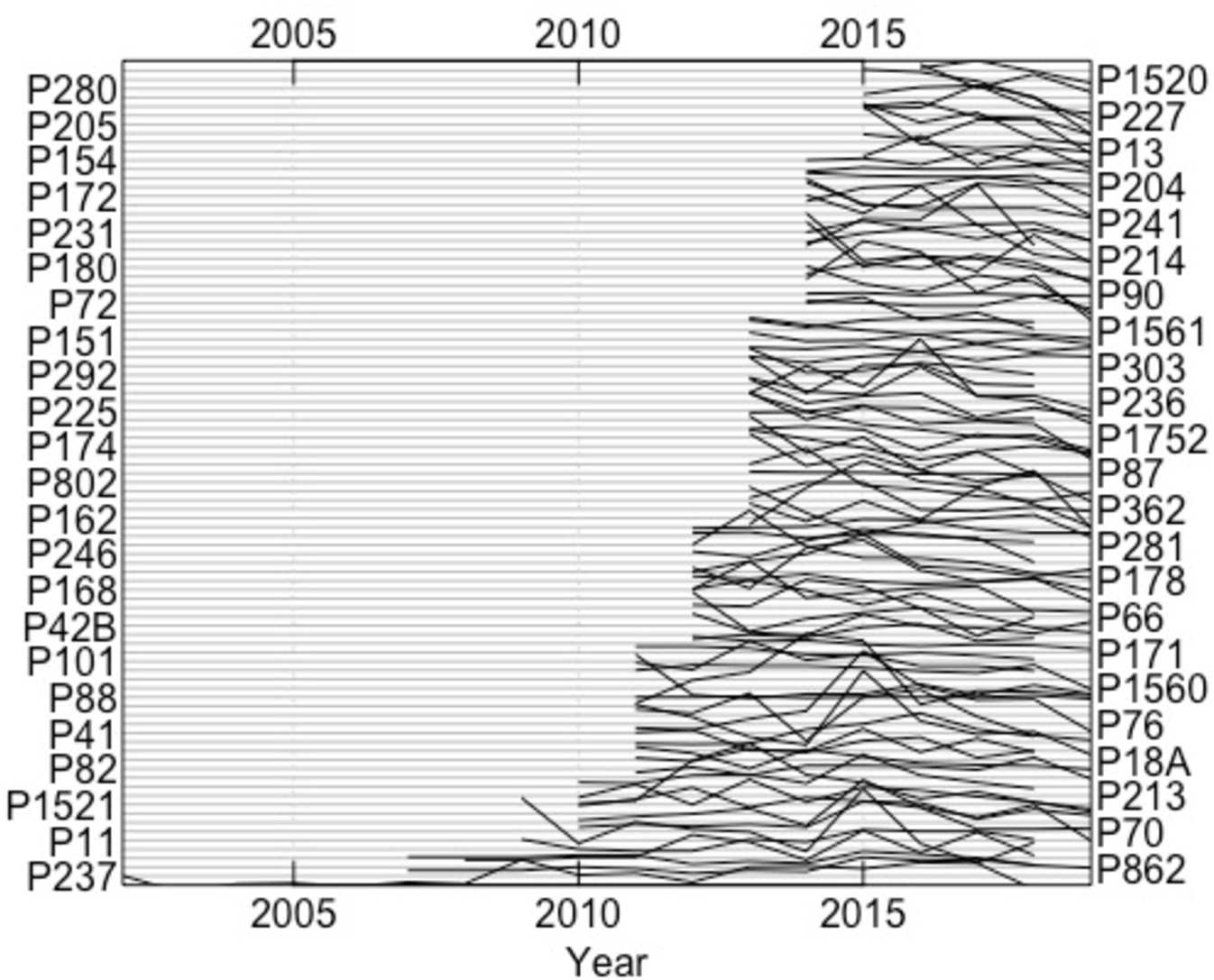

Supplement: S1 Fig — Year is represented on the horizontal axis and individual fish sample’s growth rate is represented on each vertical axis labeled by PXXX. Peaks in each growth rate represents variations of growth.The master chronology in the paper combines all of these individual growth rates into something more digestible. Only 46 of the samples are labelled due to space limitations on the Fig. (TIF) [file pone.0316247.s001.tif]

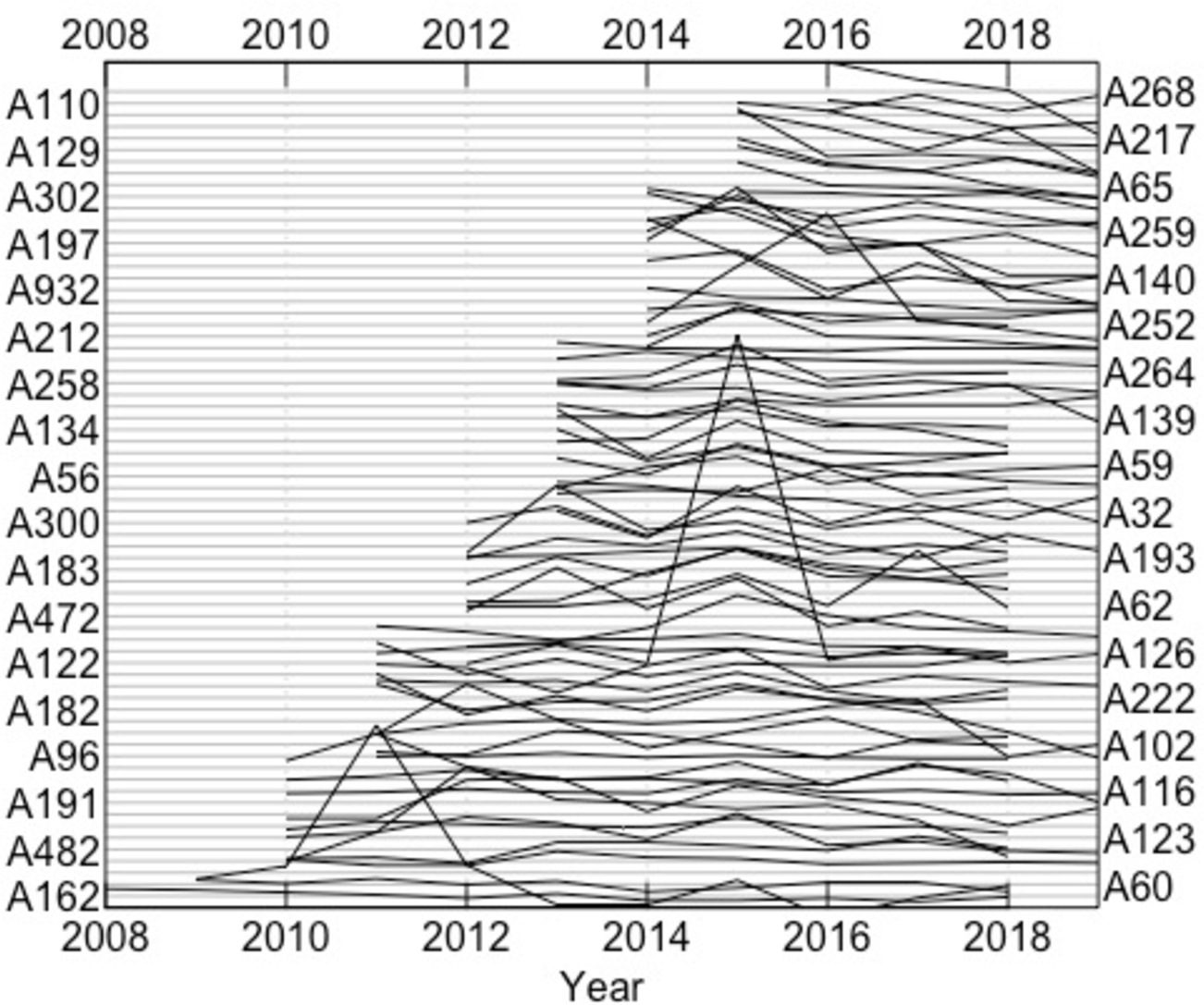

Supplement: S2 Fig — Year is represented on the horizontal axis and individual fish sample’s growth rate is represented on each vertical axis labeled by AXXX. Peaks in each growth rate represents variations of growth. The master chronology in the paper combines all of these individual growth rates into something more digestible. Only 36 samples are labelled due to the space limitations on the Fig. (TIF) [file pone.0316247.s002.tif]

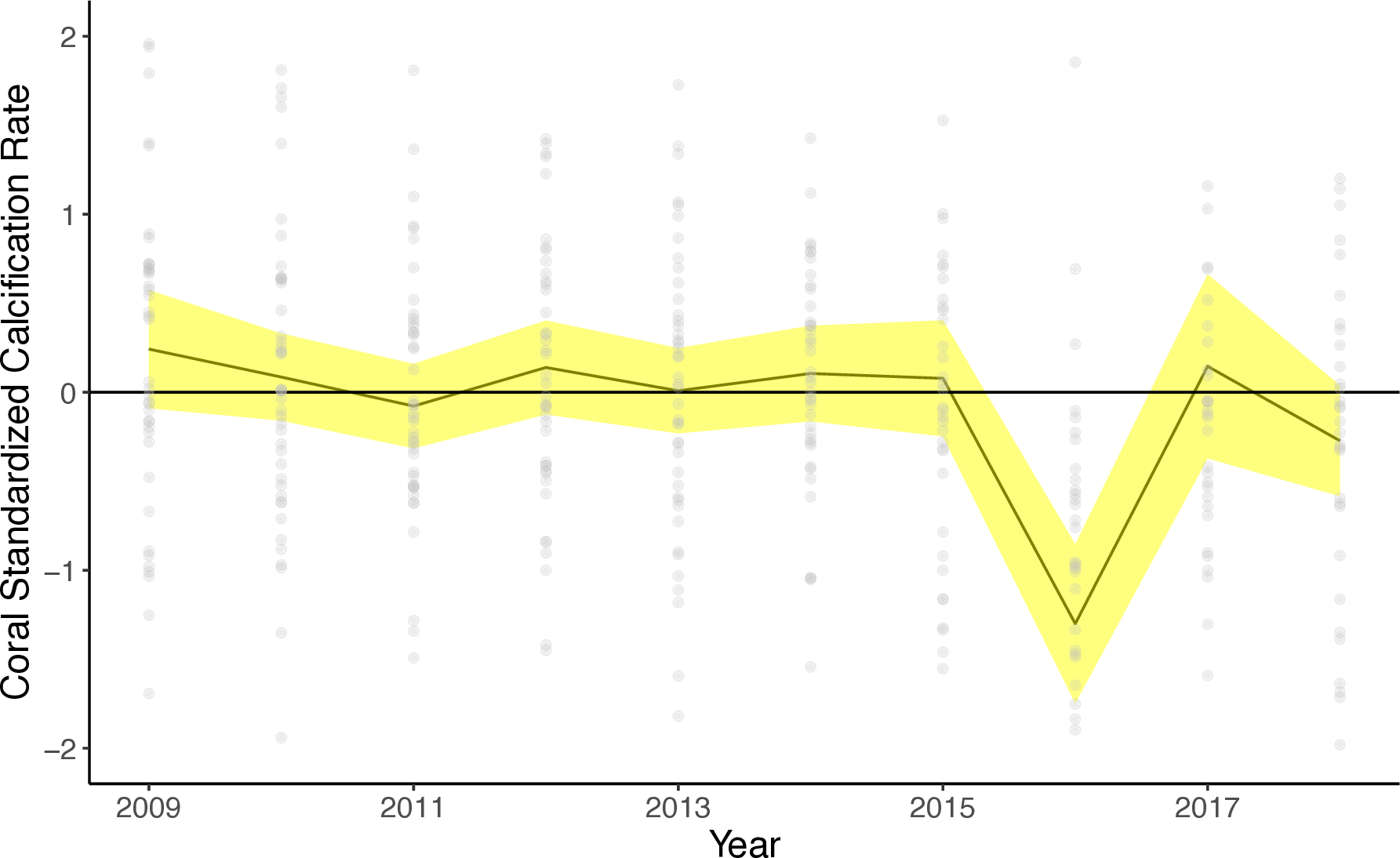

Supplement: S3 Fig — Master chronology of standardized Porites coral calcification rates from Farasan Banks between 2009 and 2018. The shaded region represents the 95% confidence interval. The grey dots represent individual sample calcification values. (TIF) [file pone.0316247.s003.tif]
