# Supplementary material for: Asynchronous effects of heat stress on growth rates of massive corals and damselfish in the Red Sea
Source: PLoS One. 2025 Jan 14;20(1):e0316247. doi: 10.1371/journal.pone.0316247 (PMC11731716; doi:10.1371/journal.pone.0316247)
Supplement: S1 Table — (PDF) [file pone.0316247.s004.pdf]

Table S1: Pearson Correlation Matrix of Predictors Used in the Marginal Models

	Year	Mean Annual Temperature	Degree Heating Weeks (DHW)	Mean Summer Sea Surface Temperature	Growth	Length	Upwelling Index (difference between June max and Aug min SST)
Year	1	0.56	-0.03	0.48	-0.28	0.014	-0.02
Mean Annual Temperature	0.56	1	0.34	0.88	-0.05	0	0.29
Degree Heating Weeks (DHW)	-0.03	0.33	1	0.64	0.30	0	0.39
Mean Summer Sea Surface Temperature	0.48	0.88	0.64	1	0.05	0	0.24
Growth	-0.28	-0.05	0.30	0.05	1	0	0.20
Length	0.01	0	0	0	0	1	0
Upwelling Index (difference between June max and Aug min SST)	-0.02	0.29	0.39	0.24	0.20	0	1
