# Supplementary material for: Asynchronous effects of heat stress on growth rates of massive corals and damselfish in the Red Sea
Source: PLoS One. 2025 Jan 14;20(1):e0316247. doi: 10.1371/journal.pone.0316247 (PMC11731716; doi:10.1371/journal.pone.0316247)
Supplement: S2 Table — Akaike’s information criterion (AIC) scores for tested Marginal Models of the damselfishes’ standardized growth rates between 2010 and 2019, listed from best to worst. All models had a repeated effect of ‘year’ with subject as individual fish samples and first-order autoregressive (AR1) as the covariance structure. To allow for comparison of models with different fixed effects, Maximum Likelihood (ML) estimation was used. (PDF) [file pone.0316247.s005.pdf]

Model Rank	Fixed Effects	AIC
1	dhw, bleachingyrs	732.2
2	dhw, bleachingyrs, upwelling	734.0
3	dhw, bleachingyrs, upwelling, yr	734.7
4	dhw, bleachingyrs, upwelling, yr, dhw X upwelling	735.5
5	dhw, bleachingyrs, upwelling, temp	735.9
6	dhw, upwelling, yr, dhw X upwelling	753.3
7	bleachingyrs, upwelling	768.9
8	bleachingyrs	775.8
9	dhw, upwelling, dhw X upwelling	869.8
10	dhw, upwelling	892.4
11	dhw	896.9
12	upwelling	984.6
13	summer	1005.2
14	temp	1005.4
15	length	1008.9
16	dxtoshore	1008.9

“bleachingyrs” = years since the bleaching event of 2015, “dhw” = degree heating weeks, “upwelling” = upwelling index, “yr” = calendar year, “summer” = mean summer temperature, “temp” = mean annual temperature, “length” = fish standard length, “dxtoshore” distance from shore.
