# Supplementary material for: Asynchronous effects of heat stress on growth rates of massive corals and damselfish in the Red Sea
Source: PLoS One. 2025 Jan 14;20(1):e0316247. doi: 10.1371/journal.pone.0316247 (PMC11731716; doi:10.1371/journal.pone.0316247)
Supplement: S3 Table — Akaike’s information criterion (AIC) scores for tested Marginal Models of the corals’ standardized growth rates between 2010 and 2018, listed from best to worst. All models had a repeated effect of ‘year’ with subject as individual coral cores and an unstructured covariance matrix. To allow for comparison of models with different fixed effects, Maximum Likelihood (ML) estimation was used. (PDF) [file pone.0316247.s006.pdf]

Model Rank	Fixed Effects	AIC
1	dhw	309.3
2	upwelling	309.6
3	dhw, upwelling	310.0
4	temp, dhw	310.8
5	temp, upwelling	310.9
6	temp	311.0
7	temp, dhw, upwelling	311.5

“dhw” = degree heating weeks, “upwelling” = upwelling index, “temp” = mean annual temperature.
